# Species Distribution and Antifungal Susceptibilities of Candida Species Isolated From Blood Culture

**DOI:** 10.7759/cureus.38183

**Published:** 2023-04-27

**Authors:** Burcu Dalyan Cilo

**Affiliations:** 1 Section of Medical Mycology, University of Health Sciences, Bursa Yuksek Ihtisas Training & Research Hospital, Bursa, TUR

**Keywords:** fungal infections, candidemia, epidemiology, candida, blood culture

## Abstract

Introduction

*Candida* species (spp.) are among the leading agents of bloodstream infections. Candidemias are a major cause of morbidity and mortality. Having an understanding of *Candida* epidemiology and antifungal susceptibility patterns in each center is crucial in guiding the management of candidemia. In this study, the species distribution and antifungal susceptibility of *Candida *spp. isolated from blood culture at the University of Health Sciences, Bursa Yuksek Ihtisas Training & Research Hospital were examined and the first data on the epidemiology of candidemia in our center were presented.

Methods

A total of 236* Candida* strains isolated from blood cultures in our hospital over a four-year period were analyzed and their antifungal susceptibilities were studied retrospectively. Strains were identified at the species complex (SC) level by the germ tube test, morphology in cornmeal-tween 80 medium, and the automated VITEK 2 Compact (bioMérieux, Marcy-l'Étoile, France) system. Antifungal susceptibility tests were performed on VITEK 2 Compact (bioMérieux, Marcy-l'Étoile, France) system. The susceptibilities of the strains to fluconazole, voriconazole, micafungin, and amphotericin B were determined according to Clinical and Laboratory Standards Institute (CLSI) guidelines and epidemiologic cut-off values.

Results

Of the *Candida (C.)* strains, 131 were *C. albicans *(55.5%), 40 were *C. parapsilosis *SC (16.9%), 21 were *C. tropicalis *(8.9%), 19 were *C. glabrata* SC (8.1%), eight were *C. lusitaniae *(3.4%), seven were *C. kefyr *(3.0%), six were *C. krusei *(2.6%), two were *C. guilliermondii *(0.8%) and two were *C. dubliniensis *(0.8%).

Amphotericin B resistance was not detected in *Candida* strains. Micafungin susceptibility was 98.3%, and four *C. parapsilosis* SC strains (10%) were intermediate (I) to micafungin. Fluconazole susceptibility was 87.2%. Apart from *C. krusei *strains which intrinsically resistant to fluconazole, three *C. parapsilosis* (7.5%), one *C. glabrata *SC (5.3%) strain were resistant (R) to fluconazole, and one *C. lusitaniae* (12.5%) strain was wild-type (WT). Voriconazole susceptibility of *Candida* strains was 98.6%. Two *C. parapsilosis* SC strains were I to voriconazole, while one strain was R.

Conclusion

In this study, the first epidemiological data of candidemia agents in our hospital were presented. It was determined that rare and naturally resistant species did not cause any problem in our center yet. *C. parapsilosis *SC strains showed decreased susceptibility to fluconazole, whereas *Candida* strains were highly susceptible to the four antifungals tested. Close monitoring of these data will help guide the treatment of candidemia.

## Introduction

*Candida* spp. are the most common cause of fungal infections [1]. Candidemias account for 50-70% of invasive *Candida* infections and *Candida *spp. rank fourth among the agents of bloodstream infections [2]. Particularly in hospitalized, immunosuppressed and critically ill patients, candidemia is a significant cause of morbidity and mortality [3,4]. Early diagnosis is important in reducing mortality and morbidity, but since it is not easy, empirical and preemptive treatment is often planned in cases with underlying risk factors and clinical findings [2,5,6]. It is important to monitor epidemiological data and antifungal susceptibility patterns at each center to initiate appropriate therapy.

*C. albicans* is the most common cause of candidemia, but an increase in infections caused by non-albicans *Candida *spp. has been observed in recent years [7-10]. In many studies, it has been reported that the spectrum of agents in candidemia varies from country to country, between years and between hospitals in the same country. For this reason, it is important to conduct surveillance studies at regular intervals in terms of the management of fungal infections in hospitals [11].

The aim of this study is to determine the species distribution and antifungal susceptibilities of Candida spp. isolated from blood samples in our hospital and to present local epidemiological data.

## Materials and methods

A total of 236 *Candida* strains isolated from blood cultures at the University of Health Sciences, Bursa Yuksek Ihtisas Training & Research Hospital over a four-year period (2018-2021) and their antifungal susceptibilities were retrospectively analyzed. The bottles incubated in a fully automated blood culture system (BACTEC FX-40, Becton Dickinson, MD, USA). Yeast cells were seen in Gram-stained microscopic examination prepared from blood culture bottles with positive signals and were inoculated onto sheep blood agar (RTA Laboratories, Kocaeli, Turkey), Eosin methylene blue (EMB) agar (RTA Laboratories, Kocaeli, Turkey), and chocolate agar (RTA Laboratories, Kocaeli, Turkey). After incubation at 37°C for 24 hours, pure culture was obtained by passaging the plates with yeast growth on Sabouraud dextrose agar (SDA) (RTA Laboratories, Kocaeli, Turkey). Strains were identified to SC level by germ tube test, morphology on cornmeal-tween 80 medium and VITEK 2 YST ID (bioMérieux, Marcy-l'Étoile, France) card on VITEK 2 Compact system (bioMérieux, Marcy-l'Étoile, France). Quality control was achieved with *Candida parapsilosis *ATCC 22019 and *Candida krusei* ATCC 6258 strains.

In the determination of antifungal susceptibility, the turbidity of *Candida* strains was set to 2.0 McFarland (1.8-2.2; DensiCheck, BioMérieux) with 0.45% sterile NaCl according to the manufacturer's instructions. They were loaded to the VITEK 2 AST YS08 fungal susceptibility card (BioMérieux) and the cards were placed into the instrument. This card includes amphotericin B (≤0.25->16 µg/ml), flucytosine (≤1->64µg/ml), fluconazole (≤0.5->64µg/ml), voriconazole (≤0.125->8µg/ml), caspofungin (≤0.125->8µg/ml) and micafungin (≤0.06->8µg/ml) [12]. In this study, flucytosine which is not available in our country and caspofungin which produced variable results in vitro susceptibility were excluded.

In the determination of antifungal susceptibility, clinical breakpoints (CBs) in Clinical and Laboratory Standards Institute (CLSI) guideline and epidemiologic cut-off values (ECOFFs) were used (Table 1) [13-14].

**Table 1:** Clinical breakpoints and epidemiologic cut-off values for Candida species ECOFFs: Epidemiologic cut-off values, CBs: Clinical breakpoints, S: Susceptible, SDD: Susceptible-dose-dependent, I: Intermediate, R: Resistant, WT: Wild-Type, NWT: Non-Wild-Type

Organism	Antifungal agent	ECOFFs (µ/ml)		CBs: (µ/ml)			
		WT	Non-WT	S	SDD	I	R
C. albicans	Amphotericin B	≤2	>2				
	Fluconazole			≤2	4		≥8
	Voriconazole			≤0.12		0.25-0.5	≥1
	Micafungin			≤0.25		0.5	≥1
*C. glabrata* SC	Amphotericin B	≤2	>2				
	Fluconazole				≤32		≥64
	Voriconazole	≤0.5	>0.5				
	Micafungin			≤0.06		0.12	≥0.25
*C. parapsilosis* SC	Amphotericin B	≤2	>2				
	Fluconazole			≤2	4		≥8
	Voriconazole			≤0.12		0.25-0.5	≥1
	Micafungin			≤2		4	≥8
C. tropicalis	Amphotericin B	≤2	>2				
	Fluconazole			≤2	4		≥8
	Voriconazole			≤0.12		0.25-0.5	≥1
	Micafungin			≤0.25		0.5	≥1
C. krusei	Amphotericin B	≤2	>2				
	Fluconazole						
	Voriconazole			≤0.5		1	≥2
	Micafungin			≤0.25		0.5	≥1
C. lusitaniae	Amphotericin B	≤2	>2				
	Fluconazole	≤2	>2				
	Voriconazole	≤0.03	>0.03				
	Micafungin	≤0.5	>0.5				
C. guilliermondii	Amphotericin B	≤2	>2				
	Fluconazole	≤8	>8				
	Voriconazole	≤0.25	>0.25				
	Micafungin	≤2	>2				
C. dubliniensis	Amphotericin B	≤2	>2				
	Fluconazole	≤0.5	>0.5				
	Voriconazole	≤0.03	>0.03				
	Micafungin	≤2	>2				
C. kefyr	Fluconazole	≤1	>1				
	Voriconazole	≤0.015	>0.015				
	Micafungin	≤0.12	>0.12				

Amphotericin B susceptibility could not be evaluated since there were no established CBs and ECOFFs for *C. kefyr*. Since the voriconazole MIC range detected by the automated antifungal susceptibility testing system we used was ≤0.125->8µg/ml, the voriconazole susceptibility of* C. kefyr* with an ECOFFs of ≤0.015 µg/ml and *C. lusitaniae* and *C. dubliniensis* strains with an ECOFFs of ≤0.03 µg/ml could not be evaluated.

## Results

When the species distribution of 236 *Candida* strains isolated from blood culture in our center over four years was examined, 131 of the isolates were *C. albicans* (55.5%), 40 of them were *C. parapsilosis* SC (16.9%), and 21 of them were *C. tropicalis* (8.9%), 19 of them were *C. glabrata* SC (8.1%), eight of them were *C. lusitaniae* (3.4%), seven of them were *C. kefyr* (3.0%), six of them were *C. krusei *(2.6%), two each of them were identified as *C. guilliermondii* (0.8%) and *C. dubliniensis *(0.8%) (Table 2).

**Table 2:** Species distribution of Candida strains

*Candida* species	2018	2019	2020	2021	TOTAL
C. albicans	29	44	22	36	131 (55.5%)
*C. parapsilosis* SC	10	13	8	9	40 (16.9%)
C. tropicalis	3	8	5	5	21 (8.9%)
*C. glabrata* SC	3	3	4	9	19 (8.1%)
C. kefyr	2	2	1	2	7 (3.0%)
C. lusitaniae	3	3	1	1	8 (3.4%)
C. krusei	1	3	2		6 (2.6%)
C. guilliermondii	1	-	-	1	2 (0.8%)
C. dubliniensis	-	2	-	-	2 (0.8%)
TOTAL	52	78	43	63	236 (100%)

The susceptibilities of the strains to fluconazole, voriconazole, micafungin and amphotericin B are presented in Table 3. *Candida* spp. were 100% WT for amphotericin B, 87.2% susceptible (S) to fluconazole, 98.6% susceptible to voriconazole and 98.3% susceptible to micafungin.

**Table 3:** Antifungal susceptibilities of Candida strains S: Susceptible, I: Intermediate, SDD: Susceptible-dose-dependent, R: Resistant, WT: Wild-Type, NWT: Non-Wild-Type *= Susceptibility could not be evaluated because the MIC range detected by the automated antifungal susceptibility testing system was above the epidemiological cut-off values. **= Could not be evaluated due to lack of established clinical breakpoint values and epidemiological cut-off values.

*Candida* species	Amphotericin B			Fluconazole			Micafungin			Voriconazole		
	S/WT	I/SDD	R/NWT	S/WT	I/SDD	R/NWT	S/WT	I/SDD	R/NWT	S/WT	I/SDD	R/NWT
*C. albicans* (n=131)	131 (100%)	0	0	130 (99.2%)	1 (0.8%)	0	131 (100%)	0	0	131 (100%)	0	0
*C. parapsilosis* SC (n=40)	40 (100%)	0	0	37 (92.5%)	0	3 (7.5%)	36 (90%)	4 (10%)	0	37 (92.5%)	1 (2.5%)	2 (5.0%)
*C. tropicalis* (n=21)	21 (100%)	0	0	21 (100%)	0	0	21 (100%)	0	0	21 (100%)	0	0
*C. glabrata* SC (n=19)	19 (100%)	0	0	0	18 (94.7%)	1 (5.3%)	19 (100%)	0	0	19 (100%)	0	0
*C. krusei* (n=6)	6 (100%)	0	0	0	0	6 (100%)	6 (100%)	0	0	6 (100%)	0	0
*C. lusitaniae *(n=8)	8 (100%)	0	0	7 (87.5%)	0	1 (12.5%)	8 (100%)	0	0	*	*	*
*C. kefyr *(n=7)	**	**	**	7 (100%)	0	0	7 (100%)	0	0	*	*	*
*C. guilliermondii* (n=2)	2 (100%)	0	0	2 (100%)	0	0	2 (100%)	0	0	2 (100%)	0	0
*C. dubliniensis* (n=2)	2 (100%)	0	0	2 (100%)	0	0	2 (100%)	0	0	*	*	*
TOTAL (n=236)	229 (100%)	0	0	206 (87.2%)	19 (8.1%)	11 (4.7%)	232 (98.3%)	4 (1.7%)	0	216 (98.6%)	1 (0.5%)	2 (0.9%)

When the antifungal susceptibilities of the species were analyzed, it was determined that all *C. albicans* strains (100%) were WT for amphotericin B and susceptible to micafungin and voriconazole. Fluconazole susceptibility was 99.2%, while one strain (0.8%) was susceptible-dose-dependent (SDD) (Table 3).

All *C. parapsilosis *SC strains were WT for amphotericin B, while micafungin susceptibility was 90% and four strains (10%) were SDD. Fluconazole susceptibility was 92.5%, three strains (7.5%) were resistant to fluconazole. Voriconazole susceptibility was 92.5%, while one strain (2.5%) was SDD and two (5.0%) were resistant (Table 3).

All *C. tropicalis* strains (100%) were susceptible to fluconazole, voriconazole, micafungin and WT for amphotericin B (Table 3).

All *C. glabrata* SC strains were susceptible to micafungin and WT for voriconazole and amphotericin B. When fluconazole susceptibility was analyzed, it was found that 94.7% of the strains were SDD to fluconazole and one strain (5.3%) was resistant (Table 3).

All *C. krusei *strains (100%) that were intrinsically resistant to fluconazole were WT for amphotericin B and susceptible to voriconazole and micafungin. All *C. kefyr* strains (100%) were WT for fluconazole and micafungin. While 87.5% of *C. lusitaniae *strains were WT for fluconazole and one strain (12.5%) was non-wild-type (NWT), 100% were WT for amphotericin B and micafungin. All C*. guilliermondii* strains (100%) were WT for fluconazole, voriconazole, amphotericin B and micafungin. All *C. dubliniensis* strains (100%) were WT for fluconazole, amphotericin B and micafungin (Table 3).

## Discussion

The incidence of candidemia has increased in recent years. However, the epidemiology of *Candida* is changing and the widespread use of antifungal agents for therapeutic and prophylactic purposes leads to an increase in the prevalence of infections caused by non-albicans species all over the world [15-18].

Among about 15 *Candida* species known to cause disease in humans, five species, *C. albicans, C. glabrata* SC, *C. parapsilosis* SC, *C. tropicalis* and *C. krusei* are responsible for 90% of infections [6].

*C. albicans* is the most commonly isolated species among candidemia agents with varying rates (37.9%-76.3%) [19-23]. Similarly, *C. albicans* (55.5%) was found to be the most common *Candida* spp. isolated in our hospital.

Among non-albicans *Candida* spp., *C. glabrata* SC is the most commonly isolated species in North America, Northern China, and Northern Europe, while *C. parapsilosis* SC ranks first in Southern Europe and Latin America [24-25]. In studies conducted in our country, *C. parapsilosis* SC has been reported as the most common species among non-albicans species with different rates (6-66%) [11, 26-32]. Consistent with this, *C. parapsilosis* SC (16.9%) was the second most frequently isolated species in our center, following *C. albicans.*

In studies from different centers,* C. tropicalis* or *C. glabrata *SC ranks second among non-albicans species [11, 26-32]. In recent years, infections caused by *C. glabrata* SC have been increasing [33]. This increase is associated with the increasing use of azoles to which *C. glabrata* SC is less susceptible [33-37]. In our study, *C. tropicalis *ranked second among non-albicans species, followed by *C. glabrata* SC.

*Candida* isolates resistant to clinically available antifungal agents are still rare, but are increasingly being reported worldwide [38-39]. Therefore, continuous monitoring of antifungal susceptibility patterns and mechanisms of resistance to clinically used agents becomes important [18].

Amphotericin B resistance is rare in *Candida* species. In a recent study, it was determined that amphotericin B susceptibility decreased over the years in *Candida* strains [30]. In another study examining 1062 *Candida* strains from different centers, amphotericin B resistance was not detected [40]. Similarly, in our study, all *Candida* strains were WT (those without mutational or acquired resistance mechanisms) for amphotericin B.

In the treatment of candidemia, echinocandins are widely used due to their broad-spectrum activity against *Candida* spp. [30]. Micafungin, one of the drugs recommended for the detection of echinocandin susceptibility, was included in our study. In the SENTRY program, low rates of echinocandin resistance were found in *Candida* strains (*C. albicans* 0.0%-0.1%, *C. parapsilosis* 0.0%-0.1%, *C. tropicalis* 0.5%-0.7%, *C. krusei* 0.0%-1.7%, *C. glabrata*​ 1.7%-3.5%) [18]. In a multicenter study in which antifungal susceptibility data of our country were presented, no echinocandin resistance was found in *Candida* strains [40]. Decreased susceptibility to echinocandins has been reported in *Candida* spp. in different studies from Latin America, Europe, and North America [41]. Likewise, in a recent study conducted in Turkey, it was revealed that echinocandin susceptibility decreased in *C. parapsilosis* SC strains over the years [30]. In our study, the echinocandin susceptibility of *Candida* strains was 98.3%, while only three *C. parapsilosis* SC strains showed decreased susceptibility to micafungin. These data emphasize the need for close monitoring of antifungal susceptibility patterns in each center to detect changes that may occur over the years.

In our study, we evaluated the susceptibility of fluconazole and voriconazole among azole group antifungal agents. Fluconazole is one of the drugs commonly used in the treatment of candidemia [42]. The fluconazole susceptibility of *Candida* strains was 87.3%. In addition to intrinsically fluconazole resistant *C. krusei* strains, three *C. parapsilosis* SC (7.5%) and one *C. glabrata* SC (5.3%) strains were resistant to fluconazole and one *C. lusitaniae* (12.5%) strain was WT.

*C. glabrata* SC is known to be less sensitive to azole drugs [42]. In studies from different countries, fluconazole resistance rates (2.8%-36.4%) are different in *C. glabrata* SC strains [43-50]. In a 20-year study involving 12 centers in our country, fluconazole resistance was found to be 0.9% in *C. glabrata* SC strains [40]. Consistent with this, only one* C. glabrata* SC strain (5.3%) was R to fluconazole in our hospital.

Fluconazole resistance in *C. parapsilosis *SC is one of the remarkable issues regarding antifungal resistance in recent years [42]. While no significant resistance was observed in strains isolated in Europe, Latin America, and Asia Pacific regions (4.6%, 4.3%, 0.6%, respectively) [18], a study conducted in South Africa found that only 37% of *C. parapsilosis *SC strains were susceptible to fluconazole [51]. In a study from our country, it was found that the susceptibility to fluconazole in *C. parapsilosis* SC strains decreased over the years (from 94% to 49%) [30]. In a study conducted in a different center in our region, fluconazole resistance was found 18.2% in *C. parapsilosis* SC [29]. In our study, fluconazole resistance was 7.5% in *C. parapsilosis* SC. Similarly, the difference in fluconazole resistance rates of *C. parapsilosis* SC strains isolated in different centers in our country was remarkable [40]. This difference is thought to be related to the clonal distribution of *C. parapsilosis* SC in which nosocomial transmission plays an important role [40].

In our study, voriconazole susceptibility in *Candida* strains was 98.6%. Two *C. parapsilosis* SC strains were found I to voriconazole and one strain R, these three strains were R to fluconazole. Cross-resistance to voriconazole can be seen in fluconazole-resistant *Candida* strains. In the SENTRY study, only 32.7% of fluconazole-resistant *C. parapsilosis* SC strains were susceptible to voriconazole [18].

The limitation of our study was that the voriconazole MIC range that could be determined by the antifungal susceptibility test system we used, was not suitable for *C. kefyr, C. lusitaniae, C. dubliniensis*. Therefore, voriconazole susceptibility could not be evaluated in *C. kefyr, C. lusitaniae, C. dubliniensis* strains. In addition, since this was a single-center study, the findings may not be valid for other centers and may also depend on local fungal epidemiology; *Candida auris*, an emerging fungal pathogen, was not evaluated in this study and further studies will be needed for this.

## Conclusions

In conclusion, this study presents the first epidemiologic data of candidemia agents in our hospital. It was determined that rare and naturally resistant species did not cause any problem in our center yet. *C. parapsilosis* SC strains showed decreased susceptibility to fluconazole, whereas* Candida *strains were highly susceptible to the four antifungals tested. It was considered that close monitoring of these data will help guide the treatment of candidemia.
